# Supplementary material for: Analysis of the mechanism of Ricinus communis L. tolerance to Cd metal based on proteomics and metabolomics
Source: PLoS One. 2023 Mar 2;18(3):e0272750. doi: 10.1371/journal.pone.0272750 (PMC9980742; doi:10.1371/journal.pone.0272750)
Supplement: S1 Table — (DOCX) [file pone.0272750.s001.docx]

Table S1 RT-qPCR primer sequence

| Name | Primer Sequence（5′-3′） | Tm（℃） |
| --- | --- | --- |
| Actin-R | GTGCTTGATTCTGGTGATGGC | 56.9 |
| Actin-F | TTGGCAGTCTCAAGTTCTTGCTC | 57.5 |
| B9RCB9-R | GGTGAGGCTTCCTTAAGAATGG | 55.7 |
| B9RCB9-F | CCTGCATCTGATCAGTCCAC | 55.6 |
| B9T1G7-R | GCTGCAATCCTGGTTCCAG | 55.9 |
| B9T1G7-F | GTCACTGGCAGTGATTCTCC | 57.0 |
| B9RXH6-R | GACTCGTGGAGTTAGCATG | 52.7 |
| B9RXH6-F | CGATCTTGTGTCTGATTCTCC | 52.9 |
| B9RTU8-R | GGTACATGTTAGCACTAGCGG | 55.6 |
| B9RTU8-F | CTCTAACATGAATAATGTACCGC | 55.2 |
| B9SIQ2-R | GACAGCCGTGCCTCAATTTC | 57.2 |
| B9SIQ2-F | GGATACGGCCATGAGTAGGC | 58.3 |
| B9SR47-R | GAAGCGTCAGGTCATGATTAGG | 55.5 |
| B9SR47-F | CAATCTCCCATTTAACTCGACCAC | 55.6 |
| B9RWI8-R | GCGCACTATTCGTGGGATGATTC | 58.7 |
| B9RWI8-F | CCTTGAGCATCAGGGATAAC | 58.5 |
| B9RGI8-R | GCACTGTGCTTTGTGTCCATG | 57.5 |
| B9RGI8-F | CAGGATCACTCCACATTAGATCGC | 57.5 |
| B9T3N2-R | GTGCGTGACAGTATGAGGATG | 55.8 |
| B9T3N2-F | GCAAACCTCTATGCCAACAC | 55.1 |
