# Supplementary material for: Analysis of the mechanism of Ricinus communis L. tolerance to Cd metal based on proteomics and metabolomics
Source: PLoS One. 2023 Mar 2;18(3):e0272750. doi: 10.1371/journal.pone.0272750 (PMC9980742; doi:10.1371/journal.pone.0272750)
Supplement: S2 Table — (DOCX) [file pone.0272750.s002.docx]

Table S2 Primer sequences of PCR and RT-qPCR

| Primer name | Primer sequence(5´-3´) |
| --- | --- |
| RcHA 4-GFP-R | ACGGGGGACTCTAGAGGATCCATGGCTAAGTCTATCAGTCTCGAAGA |
| RcHA 4-GFP-F | GCCCTTGCTCACCATGGTACCCACTGTATAATGCTGCTGAATTGTATC |
| *RcHA4*-1305.2-R | ACGGGGGACTCTTGACCATGGAGATGGCTAAGTCTATCAGTCTCGAA |
| *RcHA4*-1305.2-F | GTCATCCTTGTAATCCCATGGTTACACTGTATAATGCTGCTGAATTGT |
| atActin-R | TGGTGGAAGCACAGAAGTTG |
| atActin-F | GATCCATGTTTGGCTCCTTG |
| *RcHA4*-R | GCTGCAATCCTGGTTCCAG |
| *RcHA4*-R | GTCACTGGCAGTGATTCTCC |
