# Supplementary material for: Analysis of the mechanism of Ricinus communis L. tolerance to Cd metal based on proteomics and metabolomics
Source: PLoS One. 2023 Mar 2;18(3):e0272750. doi: 10.1371/journal.pone.0272750 (PMC9980742; doi:10.1371/journal.pone.0272750)
Supplement: S3 Table — (DOCX) [file pone.0272750.s003.docx]

Table S3 Identification Results of Differential Proteins in the Roots of ZA_VS_CK Castor Plants

| **Uniport.ID** | **Protein names** | **LOGFC** | **Pvalue** | **Regulation** |
| --- | --- | --- | --- | --- |
| B9RXH6 |  | 0.898999509 | 0.002839828 | up |
| B9R8S4 | Pentatricopeptide repeat-containing protein, | 0.49471243 | 0.007510368 | up |
| B9T1E7 | Mitochondrial oxoglutarate/malate carrier protein | 0.813076922 | 0.017466063 | up |
| B9RZK2 | Major allergen Pru ar, putative | 0.789690351 | 0.000426682 | up |
| Q9M4Q8 | Legumin B, putative (Legumin-like protein) | 0.839408517 | 0.00088968 | up |
| B9RTU8 | Basic 7S globulin 2 small subunit, putative | 0.771381122 | 0.000130233 | up |
| B9S689 | Epoxide hydrolase, putative (EC 2.7.10.2) | 0.440069357 | 0.00063584 | up |
| B9SZQ7 |  | 0.410636071 | 0.008798643 | up |
| B9SKL5 | NADH-ubiquinone oxidoreductase 39 kD subunit, putative (EC 1.6.99.3) | 0.475413038 | 0.013396593 | up |
| B9T1G7 | Plasma membrane ATPase (EC 7.1.2.1) | 0.607523485 | 0.003425406 | up |
| B9SR47 | 30S ribosomal protein S8, putative | 0.553250241 | 0.005674542 | up |
| B9SLS5 | Succinate dehydrogenase [ubiquinone] iron-sulfur subunit, mitochondrial (EC 1.3.5.1) | 0.323388313 | 0.018146424 | up |
| B9T3F2 |  | 0.366071382 | 0.001106377 | up |
| B9R730 | Epimerase domain-containing protein | 0.687818992 | 3.86E-06 | up |
| B9RH00 | Mitochondrial phosphate carrier protein, putative | 0.594770119 | 3.84E-07 | up |
| B9SPD6 | Ribosomal protein S9, putative | 0.585293888 | 0.000245291 | up |
| B9RV06 | Cytochrome C oxidase, putative | 0.341671546 | 2.54E-09 | up |
| B9SDX6 |  | 0.854787952 | 0.000416298 | up |
| B9SF35 | Legumin A, putative | 0.826166952 | 6.27E-06 | up |
| B9SE83 | 60S ribosomal protein L3, putative | 0.37049663 | 0.022992885 | up |
| B9RY91 | Alcohol dehydrogenase, putative (EC 1.1.1.195) | 0.413669299 | 0.014713357 | up |
| B9RNW9 |  | 0.334302426 | 0.008124082 | up |
| B9S038 | Ubiquinol oxidase (EC 1.10.3.11) | 0.34755471 | 0.002858318 | up |
| B9S3N5 | Cell elongation protein diminuto, putative | 0.457867345 | 0.000154766 | up |
| B9T303 | Far upstream element-binding protein, putative (EC 3.1.2.15) | 0.341186479 | 0.020260611 | up |
| Q5VKJ9 | Oleosin (Oleosin1) | 0.338041792 | 0.017148273 | up |
| B9SJ79 | Uncharacterized protein | 0.36882907 | 0.000129349 | up |
| B9RCD1 | Aquaporin PIP1.3, putative | 1.194418956 | 1.25E-05 | up |
| B9RR41 | Sucrose synthase (EC 2.4.1.13) | 0.375866902 | 0.00267317 | up |
| B9SWN0 | Heat-shock protein, putative | 0.653255474 | 0.001500548 | up |
| B9RCB9 | Receptor protein kinase, putative | 0.43086087 | 0.030514473 | up |
| B9SGV6 | Uncharacterized protein | 0.360558492 | 0.000498407 | up |
| B9RMD1 | Nuclear transport factor, putative | 0.369465326 | 0.005209731 | up |

| **Uniport.ID** | **Protein names** | **LOGFC** | **Pvalue** | **Regulation** |
| --- | --- | --- | --- | --- |
| B9RWF4 | Elongation factor 1-alpha | 0.333985139 | 0.006630827 | up |
| B9SWB1 | Aquaporin PIP2.1, putative | 0.375437253 | 0.000133699 | up |
| B9T5B5 | Heat shock protein 70 (HSP70)-interacting protein, putative | -0.342386275 | 0.045140336 | down |
| B9RYF5 | O-methyltransferase, putative (EC 2.1.1.104) | 0.350795684 | 0.005148127 | up |
| B9RHH3 | 60S ribosomal protein L6 | 0.418511363 | 0.008502266 | up |
| B9RUU3 | Protein kinase atmrk1, putative (EC 2.7.10.2) | -0.757110826 | 0.000471386 | down |
| B9SF36 | Legumin A, putative | 0.772194082 | 0.009498388 | up |
| B9RL90 | Succinate--CoA ligase [ADP-forming] subunit alpha, mitochondrial (EC 6.2.1.5) (Succinyl-CoA synthetase subunit alpha) (SCS-alpha) | -0.433423235 | 0.003586548 | down |
| B9RGI8 |  | -0.336469192 | 6.91E-10 | down |
| B9SA28 | 2S albumin, putative | 0.586173323 | 0.00450546 | up |
| B9RBM7 | Cytochrome C1, putative | 0.330853683 | 0.0015876 | up |
| B9RZ64 | Clathrin light chain | -0.418608268 | 0.019643927 | down |
| B9T1B8 | Legumin A, putative | 0.54442229 | 0.001502805 | up |
| B9RNK0 | 60S acidic ribosomal protein P1, putative | -0.55465422 | 0.041904452 | down |
| B9T3N2 | Mitochondrial import inner membrane translocase subunit tim9, putative | 0.529069505 | 0.024658451 | up |
| B9SHE7 | Tubulin alpha chain | -0.476761281 | 0.014160749 | down |
| B9S0Y9 | (S)-2-hydroxy-acid oxidase, putative (EC 1.1.3.15) | 0.404035959 | 0.013105563 | up |
| B9SU49 | Ribokinase (RK) (EC 2.7.1.15) | -0.430440084 | 0.003465428 | down |
| B9RIC4 | 40S ribosomal protein S2, putative | 0.356751754 | 0.007810359 | up |
| B9RF49 | Uncharacterized protein | 1.158060114 | 0.007718102 | up |
| B9SWB0 | 60S ribosomal protein L12, putative | -0.368462942 | 0.002433808 | down |
| B9SWX4 | Phosphatidylethanolamine binding protein, putative | 0.828650989 | 0.046953253 | up |
| B9SGI3 | Xaa-pro aminopeptidase, putative (EC 3.4.11.9) | -0.34520374 | 0.023683893 | down |
| B9S3I7 | Major latex allergen Hev b, putative | -0.566107514 | 0.000728426 | down |
| B9RHX5 |  | -0.356317814 | 0.001160487 | down |
| P07477 |  | -0.503466323 | 0.002918141 | down |
| B9R9R5 | Pectinesterase (EC 3.1.1.11) | -0.417902201 | 0.042822296 | down |
| B9RN61 | Serine/threonine-protein kinase PBS1, putative | -0.581255359 | 0.042210094 | down |
| B9TFX9 | Quinone oxidoreductase, putative (EC 1.6.5.5) | -0.363697817 | 0.034838198 | down |
| B9RC02 | Histone H4 | -0.352175369 | 0.010002788 | down |
| B9RXX3 | Flavoprotein wrbA, putative | -0.517129245 | 0.030074572 | down |
| B9RXB8 | Homogentisate 1,2-dioxygenase, putative (EC 1.13.11.5) | -0.536929804 | 0.000228336 | down |
| B9SB31 | Ferredoxin--NADP reductase, chloroplastic (FNR) | -0.331865596 | 0.042448223 | down |
| B9RHE0 | NADH dehydrogenase, putative | -0.344454964 | 7.70E-06 | down |
| B9T2C1 | Uncharacterized protein | -0.64333342 | 0.04418976 | down |
| P04264 |  | -0.336001231 | 0.005104668 | down |

| **Uniport.ID** | **Protein names** | **LOGFC** | **Pvalue** | **Regulation** |
| --- | --- | --- | --- | --- |
| B9S2B6 | Voltage-dependent anion-selective channel, putative | -0.591413542 | 0.005961973 | down |
| B9T5E6 | Legumin B, putative | 0.526521332 | 0.033342302 | up |
| B9R846 | Histone H3 | 0.352254056 | 0.0367268 | up |
| B9S4D6 |  | 0.541365406 | 0.040549914 | up |
| B9RWV1 | Cytochrome B5 isoform 1, putative (EC 1.7.1.1) | 0.323372475 | 0.037134746 | up |
| P35527 |  | -0.386008451 | 0.009413003 | down |
