# Supplementary material for: Analysis of the mechanism of Ricinus communis L. tolerance to Cd metal based on proteomics and metabolomics
Source: PLoS One. 2023 Mar 2;18(3):e0272750. doi: 10.1371/journal.pone.0272750 (PMC9980742; doi:10.1371/journal.pone.0272750)
Supplement: S4 Table — (DOCX) [file pone.0272750.s004.docx]

Table S4 Identification Results of Differential Proteins in the Roots of ZB_VS_CK Castor Plants

| **Uniport.ID** | **Protein names** | **LOGFC** | **Pvalue** | **Regulation** |
| --- | --- | --- | --- | --- |
| B9RZX8 | Peroxiredoxin, putative (EC 1.11.1.7) | 1.484328049 | 0.017090028 | up |
| B9SF37 | Legumin A, putative | 1.171467166 | 0.004854948 | up |
| Q5VKJ8 | Oleosin | 0.861871218 | 0.02175514 | up |
| B9SA28 | 2S albumin, putative | 1.417533708 | 7.26E-06 | up |
| B9SPD6 | Ribosomal protein S9, putative | 0.802089866 | 0.013714139 | up |
| B9SA39 | Thiamine thiazole synthase, chloroplastic (Thiazole biosynthetic enzyme) (EC 2.4.2.60) | 0.565384702 | 0.018997519 | up |
| B9S250 | ATP binding protein, putative | 0.678179855 | 0.002493682 | up |
| B9SDX6 |  | 1.265085074 | 0.01625191 | up |
| B9SA35 | Sweet protein mabinlin-1 chain A (Sweet protein mabinlin-1, chain B) | 1.570363366 | 8.26E-06 | up |
| B9SQ85 | Uncharacterized protein | 1.06435794 | 0.001179756 | up |
| B9T1B8 | Legumin A, putative | 1.072178747 | 0.001508705 | up |
| B9RWT3 | Ammonium transporter | 0.636028565 | 0.030986884 | up |
| B9T5E7 | Glutelin type-A 3, putative | 1.262234141 | 7.33E-05 | up |
| B9SF35 | Legumin A, putative | 1.153269093 | 0.000373038 | up |
| B9RTM9 | Vicilin GC72-A, putative | 0.621219351 | 0.000971306 | up |
| B9RPJ9 | 40S ribosomal protein S4 | 0.524678778 | 0.03813157 | up |
| B9T9Z4 | Uncharacterized protein | 0.406206447 | 0.003563671 | up |
| B9RA61 | Copine, putative | 0.419259045 | 0.028703569 | up |
| B9RY06 | Uncharacterized protein | 0.600099299 | 0.002981587 | up |
| B9SGV6 | Uncharacterized protein | 0.495333218 | 0.035883662 | up |
| B9SN80 |  | 1.734741376 | 0.000174838 | up |
| B9SIQ3 | Miraculin, putative | 0.65272549 | 0.038647079 | up |
| B9S5Z8 | Secretory carrier-associated membrane protein | 0.324679632 | 0.022254601 | up |
| B9RQT8 | Heat-shock protein, putative | 0.74443704 | 0.00016492 | up |
| B9T1G7 | Plasma membrane ATPase (EC 7.1.2.1)（质膜ATPase） | 0.532432232 | 0.042125828 | up |
| B9SIQ2 | Alpha-amylase/subtilisin inhibitor, putative | 0.423807709 | 0.00943068 | up |
| B9SF36 | Legumin A, putative | 1.145402682 | 0.000195387 | up |
| B9RRQ8 | Protein YME1, putative (EC 3.6.4.3) | 0.541323785 | 0.038238263 | up |
| G1D766 | Ribulose bisphosphate carboxylase large chain (RuBisCO large subunit) (EC 4.1.1.39) | 1.079393411 | 0.003240362 | up |
| B9RWM3 | Potassium channel beta, putative (EC 1.1.1.91) | 0.348000478 | 0.003785423 | up |
| B9SAZ1 | Reticuline oxidase, putative (EC 1.21.3.3) | 0.384049807 | 0.004828375 | up |
| B9RA25 | Superoxide dismutase [fe], putative (EC 1.15.1.1) | 0.446736944 | 0.000477365 | up |
| B9RWI8 | 60S ribosomal protein L13a, putative | 0.542581281 | 0.049686674 | up |
| B9SB56 |  | 0.596427687 | 0.009749667 | up |

| **Uniport.ID** | **Protein names** | **LOGFC** | **Pvalue** | **Regulation** |
| --- | --- | --- | --- | --- |

| B9RTU6 | Basic 7S globulin 2 small subunit, putative | 0.405430172 | 3.97E-06 | up |
| --- | --- | --- | --- | --- |
| B9SA37 | 2S sulfur-rich seed storage protein large subunit, putative | 0.936723424 | 0.007599281 | up |
| B9T7V2 | Spermidine synthase 1, putative (EC 2.1.1.53) | 0.347032599 | 0.014651162 | up |
| B9RD54 | Uncharacterized protein | 0.373296486 | 0.023829873 | up |
| B9RXH6 |  | 0.600192864 | 0.003529377 | up |
| B9RIS9 | 40S ribosomal protein S10, putative | 0.454411738 | 0.002610596 | up |
| Q5VKJ9 | Oleosin (Oleosin1) | 0.434930925 | 0.006317319 | up |
| B9RNW9 |  | 0.352294699 | 0.000964496 | up |
| Q70AP4 | Aquaporin (Aquaporin PIP1.3, putative) | 0.664376997 | 0.000709397 | up |
| B9SQP0 | Embryonic abundant protein, putative | 1.121223041 | 0.012159588 | up |
| B9SWN0 | Heat-shock protein, putative | 0.757350259 | 2.88E-06 | up |
| B9S543 | Vesicle-associated membrane protein, putative | 0.445346034 | 0.008567084 | up |
| B9RWM4 | Cytochrome B5 isoform 1, putative (EC 1.7.1.1) | 0.356620813 | 0.000163392 | up |
| B9SR47 | 30S ribosomal protein S8, putative | 0.405827797 | 0.002406131 | up |
| B9SA32 | 2S albumin, putative | 0.794188674 | 0.004469994 | up |
| Q9M4Q8 | Legumin B, putative (Legumin-like protein) | 0.938716608 | 0.023410238 | up |
| B9RF47 | Short chain dehydrogenase, putative (EC 1.1.1.100) | 0.834460152 | 0.000234107 | up |
| B9T3N2 | Mitochondrial import inner membrane translocase subunit tim9, putative | 0.771388706 | 0.003185701 | up |
| B9RTU8 | Basic 7S globulin 2 small subunit, putative | 0.521359784 | 2.22E-05 | up |
| B9S0Y9 | (S)-2-hydroxy-acid oxidase, putative (EC 1.1.3.15) | 0.404412396 | 4.38E-08 | up |
| B9T5E6 | Legumin B, putative | 0.952953473 | 2.36E-06 | up |
| B9T266 | C-CAP/cofactor C-like domain-containing protein | -0.346788846 | 0.001247683 | down |
| B9RF49 | Uncharacterized protein | 1.321526733 | 0.026487997 | up |
| B9RUU3 | Protein kinase atmrk1, putative (EC 2.7.10.2) | -0.598549128 | 2.69E-05 | down |
| B9R897 | Tubulin beta chain | -0.446777228 | 0.002460734 | down |
| B9SBM0 | 60S ribosomal protein L9, putative | -0.538018999 | 0.045304937 | down |
| B9RTB8 | Major allergen Pru ar, putative | -0.410140086 | 0.008513153 | down |
| B9SS29 | ATP synthase delta chain, mitochondrial, putative (EC 3.6.3.14) | 1.004394742 | 0.029812613 | up |
| B9S6H9 | 40S ribosomal protein S12 | -0.360424808 | 0.002498174 | down |
| B9SZY7 | Hsp70-binding protein, putative | -0.384821255 | 1.02E-05 | down |
| B9RGI8 |  | -0.414405154 | 2.08E-08 | down |
| B9S3I7 | Major latex allergen Hev b, putative | -0.461014048 | 0.001974369 | down |
| B9SHE7 | Tubulin alpha chain | -0.525725762 | 0.009853301 | down |
| B9R748 | WPP domain-containing protein | -0.422320175 | 0.001093491 | down |
| B9T1I6 | Protein sco1, putative (EC 3.4.21.43) | -0.388930285 | 0.009762113 | down |
| B9SZ80 | Calcium ion binding protein, putative | -0.41212414 | 0.00829302 | down |
|  |  |  |  |  |

| **Uniport.ID** | **Protein names** | **LOGFC** | **Pvalue** | **Regulation** |
| --- | --- | --- | --- | --- |

| B9RZD3 | 4-hydroxy-3-methylbut-2-enyl diphosphate reductase, putative (EC 1.17.1.2) | 0.357254003 | 8.40E-05 | up |
| --- | --- | --- | --- | --- |
| B9S1P7 | Pectin acetylesterase (EC 3.1.1.-) | -0.385171844 | 0.001946811 | down |
| B9RWL7 | 3-ketoacyl-CoA thiolase B, putative (EC 2.3.1.16) | -0.566221742 | 0.028177435 | down |
| B9RGS5 | Eukaryotic peptide chain release factor subunit, putative | -0.423725401 | 0.000248991 | down |
| B9SPY1 | Protein-lysine N-methyltransferase RCOM_0466760 (EC 2.1.1.-) | -0.391453302 | 2.35E-05 | down |
| B9RXS2 | Peroxidase (EC 1.11.1.7) | -0.52905376 | 0.00881189 | down |
| B9RZ64 | Clathrin light chain | -0.501268377 | 0.00902856 | down |
| B9S447 | TAXi_N domain-containing protein | 0.684625741 | 0.01573921 | up |
| B9RWQ3 | NADH dehydrogenase, putative (EC 1.6.99.3) | -0.418425434 | 0.000557942 | down |
| B9RPS9 | Peroxidase (EC 1.11.1.7) | -0.521822912 | 0.000276573 | down |
| P07477 |  | -0.49623717 | 0.001293131 | down |
| B9RPT0 | Peroxidase (EC 1.11.1.7) | -0.342805309 | 0.004082241 | down |
| B9RT36 |  | -0.526455423 | 0.041204682 | down |
| B9T394 | Endomembrane-associated protein, putative | -0.469523724 | 0.014770009 | down |
| B9S8E1 | Preprotein translocase secy subunit, putative | 0.600975779 | 0.014568845 | up |
| B9RTC8 | Major allergen Pru ar, putative | -0.373809995 | 0.015543513 | down |
| B9S875 | Actin, putative | -0.331504097 | 0.008526768 | down |
| P04264 |  | -0.384292297 | 0.001944376 | down |
| B9T7Z4 | Kelch domain-containing protein, putative | -0.415546297 | 0.012520867 | down |
| B9RM00 | Beta-hexosaminidase (EC 3.2.1.52) | -0.585141329 | 0.001162429 | down |
| B9SH74 | Aldehyde dehydrogenase, putative (EC 1.2.1.36) | -0.393270574 | 0.004366063 | down |
| B9RHE0 | NADH dehydrogenase, putative | -0.369723606 | 7.18E-06 | down |
| B9RC02 | Histone H4 | -0.478896103 | 0.010368367 | down |
| B9SWV6 | Aldose 1-epimerase (Galactose mutarotase) | -0.502163805 | 0.009708734 | down |
| B9T180 | Germin-like protein | 0.531278911 | 0.001848441 | up |
| B9RTE0 | N-carbamoyl-L-amino acid hydrolase, putative | -0.602715424 | 0.005362864 | down |
| P35527 |  | -0.680084032 | 0.001449776 | down |
| B9RYN7 | 26S proteasome non-atpase regulatory subunit | -1.561561433 | 0.035070445 | down |
