# Supplementary material for: Analysis of the mechanism of Ricinus communis L. tolerance to Cd metal based on proteomics and metabolomics
Source: PLoS One. 2023 Mar 2;18(3):e0272750. doi: 10.1371/journal.pone.0272750 (PMC9980742; doi:10.1371/journal.pone.0272750)
Supplement: S5 Table — (DOCX) [file pone.0272750.s005.docx]

Table S5 Identification Results of Differential Proteins in the Roots of ZC_VS_CK Castor Plants

| **Uniport.ID** | **Protein names** | **LOGFC** | **Pvalue** | **Regulation** |
| --- | --- | --- | --- | --- |
| B9RTB8 | Major allergen Pru ar, putative | -0.495312975 | 0.006811352 | down |
| B9RBN7 | Prohibitin, putative | 0.493303181 | 0.013008465 | up |
| B9RHX5 |  | 0.348363398 | 0.000102086 | up |
| B9SZ67 | Wound-induced protein WIN1, putative | 0.515828093 | 0.002466631 | up |
| B9RZR3 | Uncharacterized protein | 0.376064866 | 0.000754719 | up |
| B9RSX8 | Peroxidase (EC 1.11.1.7) | 0.334410323 | 0.013209683 | up |
| B9RTU8 | Basic 7S globulin 2 small subunit, putative | 0.765310643 | 6.65E-05 | up |
| B9SIQ3 | Miraculin, putative | 0.810571635 | 1.16E-05 | up |
| B9RY91 | Alcohol dehydrogenase, putative (EC 1.1.1.195) | 0.354437861 | 0.041680845 | up |
| B9SIQ2 | Alpha-amylase/subtilisin inhibitor, putative | 0.494044701 | 0.003819292 | up |
| B9RCT8 | UDP-glucose 4-epimerase, putative (EC 5.1.3.2) | 0.392960885 | 0.002898429 | up |
| B9ST23 | Uncharacterized protein | 0.482999338 | 0.000301212 | up |
| B9SD83 | Cinnamate 4-hydroxylase, putative (EC 1.14.13.11) | 0.59193665 | 0.004429262 | up |
| B9S5W1 | Zeta-coat protein, putative | 0.594114879 | 0.012504287 | up |
| B9RMD1 | Nuclear transport factor, putative | 0.508819646 | 0.001651473 | up |
| B9RWI8 | 60S ribosomal protein L13a, putative | 0.650792237 | 3.25E-05 | up |
| B9RWT3 | Ammonium transporter | 0.401315872 | 0.02240008 | up |
| B9RT03 | CASP-like protein 1D1 (RcCASPL1D1) | 0.427532307 | 0.002297375 | up |
| B9RMI6 | NADH dehydrogenase [ubiquinone] flavoprotein 1, mitochondrial (EC 1.6.99.3) (EC 7.1.1.2) | 0.562027479 | 0.046335084 | up |
| B9SR47 | 30S ribosomal protein S8, putative | 0.376780438 | 0.009121203 | up |
| B9SE07 | Eukaryotic translation initiation factor 3 RNA-binding subunit, putative | 0.470808471 | 0.038284269 | up |
| B9SAY9 | Reticuline oxidase, putative (EC 1.21.3.3) | 0.367033461 | 6.10E-05 | up |
| B9T1R1 | Uncharacterized protein | 0.322404625 | 0.005048396 | up |
| B9RA25 | Superoxide dismutase [fe], putative (EC 1.15.1.1) | 0.366702777 | 0.046204947 | up |
| B9SZY7 | Hsp70-binding protein, putative | -0.331957545 | 6.45E-06 | down |

| **Uniport.ID** | **Protein names** | **LOGFC** | **Pvalue** | **Regulation** |
| --- | --- | --- | --- | --- |
| B9S0Y9 | (S)-2-hydroxy-acid oxidase, putative (EC 1.1.3.15) | 0.492148277 | 0.006545237 | up |
| B9S228 | Nucleic acid binding protein, putative | -0.328489084 | 2.61E-05 | down |
| B9SHE7 | Tubulin alpha chain | -0.400435006 | 0.027178318 | down |
| P35527 |  | 0.497761449 | 0.000962412 | up |
| B9T266 | C-CAP/cofactor C-like domain-containing protein | -0.355772541 | 0.002449713 | down |
| Q70AP4 | Aquaporin (Aquaporin PIP1.3, putative) | 0.323716375 | 0.000278031 | up |
| B9S8E1 | Preprotein translocase secy subunit, putative | 0.39994435 | 0.028854159 | up |
| B9R897 | Tubulin beta chain | -0.365877887 | 0.011221339 | down |
| B9RUU3 | Protein kinase atmrk1, putative (EC 2.7.10.2) | -0.726102455 | 7.07E-05 | down |
| B9RFB0 | Cytochrome c, putative | -0.456985681 | 0.033858065 | down |
| B9SIQ4 | Miraculin, putative | 0.867426937 | 0.047831627 | up |
| B9RC02 | Histone H4 | -0.367119515 | 0.001919515 | down |
| B9RM00 | Beta-hexosaminidase (EC 3.2.1.52) | -0.45247456 | 0.002585729 | down |
| B9RWL7 | 3-ketoacyl-CoA thiolase B, putative | -0.566542804 | 0.024381929 | down |
| B9S2B7 | Protein PPLZ12, putative | 0.350765703 | 0.008815516 | up |
| B9SWB0 | 60S ribosomal protein L12, putative | -0.436343509 | 5.43E-05 | down |
| B9SRL5 | 60S ribosomal protein L24, putative | -0.343275927 | 0.030783845 | down |
