# Supplementary material for: Analysis of the mechanism of Ricinus communis L. tolerance to Cd metal based on proteomics and metabolomics
Source: PLoS One. 2023 Mar 2;18(3):e0272750. doi: 10.1371/journal.pone.0272750 (PMC9980742; doi:10.1371/journal.pone.0272750)
Supplement: S6 Table — (DOCX) [file pone.0272750.s006.docx]

| **number** | | **Compounds** | **VIP** | **Fold_Change** | | **Type** |
| --- | --- | --- | --- | --- | --- | --- |
|  | Kaempferol-3-O-glucoside-7-O-rhamnoside | | 1.01E+00 | | 3.99E+00 | up |
|  | Myristic Acid | | 1.40E+00 | | 4.78E-01 | down |
|  | 2-Methoxybenzoic acid | | 1.42E+00 | | 8.94E-02 | down |
|  | Betaine | | 1.43E+00 | | 4.00E+00 | up |
|  | L-AsparticAcid | | 1.42E+00 | | 3.75E-01 | down |
|  | Anchoic Acid | | 1.40E+00 | | 4.39E-01 | down |
|  | SubericAcid | | 1.42E+00 | | 4.28E-01 | down |
|  | Lactose | | 1.33E+00 | | 4.99E-01 | down |
|  | D-Arabitol | | 1.37E+00 | | 2.79E+00 | up |
|  | L-Arabitol | | 1.43E+00 | | 5.02E+03 | up |
|  | 3-Hydroxybutyrate | | 1.38E+00 | | 4.40E-01 | down |
|  | 9-(β-D-Arabinofuranosyl)hypoxanthine Naringenin | | 1.38E+00 | | 2.30E+00 | up |
|  | chalcone(4,2',4',6'-Tetrahydroxychalcone) | | 1.41E+00 | | 2.43E-01 | down |
|  | Mannitol | | 1.22E+00 | | 3.06E-01 | down |
|  | Aldehydo-D-galacturonate | | 1.40E+00 | | 4.66E-01 | down |
|  | Caffeine | | 1.42E+00 | | 9.47E-02 | down |
|  | Turanose | | 1.34E+00 | | 2.72E+00 | up |
|  | MAG(18:2)isomer1 | | 1.03E+00 | | 3.93E-01 | down |
|  | MAG(18:1)isomer2 | | 1.37E+00 | | 4.34E-01 | down |
|  | MAG(18:4)isomer3 | | 1.34E+00 | | 4.94E-01 | down |
|  | Cocamidopropyl βine | | 1.42E+00 | | 2.18E-01 | down |
|  | MAG(18:3)isomer2 | | 1.36E+00 | | 3.90E-01 | down |
|  | MAG(18:1)isomer1 | | 1.32E+00 | | 3.55E-01 | down |
|  | Lauric acid | | 1.42E+00 | | 3.94E-01 | down |
|  | Syringic acid O-glucoside | | 1.11E+00 | | 2.05E+00 | up |
|  | L-Glutamic acid | | 1.39E+00 | | 3.68E-01 | down |
|  | L-(+)-Lysine | | 1.20E+00 | | 4.03E-01 | down |
|  | L-Glutamine | | 1.42E+00 | | 3.31E-01 | down |
|  | Adenosine | | 1.40E+00 | | 2.20E+00 | up |
|  | Methyl gallate | | 1.32E+00 | | 4.76E-01 | down |
|  | Ethyl gallate | | 1.31E+00 | | 3.46E-01 | down |
|  | D-(+)-Sucrose | | 1.34E+00 | | 2.50E+00 | up |
|  | Gluconic acid | | 1.41E+00 | | 3.57E-01 | down |
|  | Pantothenol | | 1.42E+00 | | 8.79E-02 | down |
|  | 1-O-Galloyl-β-D-glucose | | 1.40E+00 | | 3.98E+00 | up |
|  | 1,2,3,4,6-Penta-O-galloyl-β-D-glucose | | 1.29E+00 | | 2.16E+00 | up |
|  | Pentagalloylglucose Isorhamnetin | | 1.21E+00 | | 2.04E+00 | up |

Table S6 Identification Results of Differential Metabolites in the Roots of ZA_VS_CK Castor Plants

| **number** | | **Compounds** | **VIP** | **Fold_Change** | | **Type** |
| --- | --- | --- | --- | --- | --- | --- |
|  | 3-O-β-(2''-O-acetyl-β-D-glucuronide) | | 1.15E+00 | | 4.66E+00 | up |
|  | Quercetin-7-O-(6'-O-malonyl)-β-D-glucoside | | 1.35E+00 | | 3.21E+00 | up |
|  | 5-Aminocycloheptane-1,2,3-triol | | 1.42E+00 | | 3.35E+00 | up |
