# Supplementary material for: Analysis of the mechanism of Ricinus communis L. tolerance to Cd metal based on proteomics and metabolomics
Source: PLoS One. 2023 Mar 2;18(3):e0272750. doi: 10.1371/journal.pone.0272750 (PMC9980742; doi:10.1371/journal.pone.0272750)
Supplement: S7 Table — (DOCX) [file pone.0272750.s007.docx]

| **number** | | **Compounds** | | **VIP** | | **Fold_Change** | | **Type** |
| --- | --- | --- | --- | --- | --- | --- | --- | --- |
|  | Kaempferol-3-O-glucoside-7-O-rhamnoside | | | 1.29E+00 | | | 3.29E+00 | up |
|  | Ursolic acid-OCH3 | | | 1.34E+00 | | | 2.11E+00 | up |
|  | Myristic Acid | | | 1.36E+00 | | | 3.83E-01 | down |
|  | Choline alfoscerate | | | 1.28E+00 | | | 2.83E+00 | up |
|  | 2-Methoxybenzoic acid | | | 1.22E+00 | | | 4.16E-01 | down |
|  | 2-Furanoic acid | | | 1.31E+00 | | | 3.24E-01 | down |
|  | Betaine | | | 1.37E+00 | | | 4.20E+00 | up |
|  | Ribitol | | | 1.29E+00 | | | 2.55E+00 | up |
|  | L-AsparticAcid | | | 1.37E+00 | | | 3.01E-01 | down |
|  | Anchoic Acid | | | 1.35E+00 | | | 4.23E-01 | down |
|  | SubericAcid | | | 1.36E+00 | | | 3.96E-01 | down |
|  | L-(+)-Tartaric acid | | | 1.34E+00 | | | 4.29E-01 | down |
|  | L-PyroglutamicAcid | | | 1.33E+00 | | | 4.81E-01 | down |
|  | Pipecolinic acid | | | 1.30E+00 | | | 4.56E-01 | down |
|  | Lactose | | | 1.28E+00 | | | 4.19E-01 | down |
|  | Elaidic Acid | | | 1.35E+00 | | | 4.99E-01 | down |
|  | D-Arabitol | | | 1.34E+00 | | | 3.76E+00 | up |
|  | L-Arabitol | | | 1.38E+00 | | | 4.03E+03 | up |
|  | 3-Hydroxybutyrate | | | 1.34E+00 | | | 3.78E-01 | down |
|  | 3'-Aenylic acid | | | 1.15E+00 | | | 4.25E-01 | down |
|  | 9-(β-D-Arabinofuranosyl)hypoxanthine | | | 1.24E+00 | | | 2.29E+00 | up |
|  | Naringenin chalcone(4,2',4',6'-Tetrahydroxychalcone) | | | 1.23E+00 | | | 3.59E-01 | down |
|  | Aldehydo-D-galacturonate | | | 1.34E+00 | | | 2.91E-01 | down |
|  | Stearic Acid | | | 1.35E+00 | | | 4.46E-01 | down |
|  | Caffeine | | | 1.31E+00 | | | 1.18E-01 | down |
|  | 11-Octadecanoic acid(Vaccenic acid) | | | 1.35E+00 | | | 4.88E-01 | down |
|  | Oxidized Glutathione | | | 1.36E+00 | | | 1.82E-01 | down |
|  | Isomaltulose | | | 1.35E+00 | | | 2.50E-01 | down |
|  | MAG(18:2)isomer1 | | | 1.19E+00 | | | 4.76E-01 | down |
|  | MAG(18:3)isomer3 | | | 1.20E+00 | | | 3.94E-01 | down |
|  | Cocamidopropyl βine | | 1.30E+00 | | | | 2.85E-01 | down |
|  | Lauric acid | | | | 1.35E+00 | | 3.01E-01 | down |
|  | L-Glutamic acid | | | 1.36E+00 | | | 1.10E-01 | down |
|  | L-(+)-Lysine | | | 1.14E+00 | | | 3.95E-01 | down |
|  | L-Glutamine | | | 1.32E+00 | | | 3.59E-01 | down |
|  | 2,6-Diaminooimelic acid | | | 1.31E+00 | | | 2.07E+00 | up |
|  | Ethyl gallate | | | 1.25E+00 | | | 3.34E-01 | down |
|  | Kaempferol 3-O-rutinoside(Nicotiflorin) | | | 1.23E+00 | | | 3.03E+00 | up |

Table S7 Identification Results of Differential Metabolites in the Roots of ZB_VS_CK Castor Plants

| **number** | | **Compounds** | **VIP** | **Fold_Change** | | **Type** |
| --- | --- | --- | --- | --- | --- | --- |
|  | Gluconic acid | | 1.35E+00 | | 2.64E-01 | down |
|  | Pantothenol | | 1.36E+00 | | 3.78E-01 | down |
|  | Kaempferol 3-O-robinobioside(Biorobin) | | 1.28E+00 | | 3.21E+00 | up |
|  | 2-Hydroxybutanoic acid | | 1.34E+00 | | 4.35E-01 | down |
|  | 4-Hydroxy-L-glutamic acid | | 1.32E+00 | | 2.34E+00 | up |
|  | 1-O-Galloyl-β-D-glucose | | 1.33E+00 | | 2.73E+00 | up |
|  | 1,2,3,4,6-Penta-O-galloyl-β-D-glucose | | 1.14E+00 | | 4.93E-01 | down |
|  | Pentagalloylglucose Isorhamnetin | | 1.30E+00 | | 4.51E-01 | down |
|  | 3-O-β-(2''-O-acetyl-β-D-glucuronide) | | 1.25E+00 | | 3.19E+00 | up |
|  | 1-Feruloyl-sn-glycerol | | 1.21E+00 | | 2.16E+00 | up |
|  | 2-Feruloyl-sn-glycerol | | 1.28E+00 | | 2.76E+00 | up |
|  | Isoquercitrin(Quercetin 3-O-β-D-glucoside) | | 1.05E+00 | | 2.92E+00 | up |
|  | Quercetin-7-O-(6'-O-malonyl)-β-D-glucoside | | 1.22E+00 | | 3.76E+00 | up |
|  | 5-Aminocycloheptane-1,2,3-triol | | 1.32E+00 | | 3.74E+00 | up |
