# Supplementary material for: Analysis of the mechanism of Ricinus communis L. tolerance to Cd metal based on proteomics and metabolomics
Source: PLoS One. 2023 Mar 2;18(3):e0272750. doi: 10.1371/journal.pone.0272750 (PMC9980742; doi:10.1371/journal.pone.0272750)
Supplement: S8 Table — (DOCX) [file pone.0272750.s008.docx]

Table S8 Identification Results of Differential Metabolites in the Roots of ZC_VS_CK Castor Plants

| **number** | **Compounds** | **VIP** | **Fold_Change** | | **Type** |
| --- | --- | --- | --- | --- | --- |
|  | L-Asparagine Anhydrous | 1.13E+00 | | 3.77E-01 | down |
|  | Myristic Acid | 1.31E+00 | | 2.99E-01 | down |
|  | 2-Methoxybenzoic acid | 1.32E+00 | | 1.92E-01 | down |
|  | 2-Furanoic acid | 1.22E+00 | | 3.01E-01 | down |
|  | Betaine | 1.32E+00 | | 4.26E+00 | up |
|  | L-AsparticAcid | 1.32E+00 | | 2.35E-01 | down |
|  | Anchoic Acid | 1.29E+00 | | 3.58E-01 | down |
|  | SubericAcid | 1.29E+00 | | 3.11E-01 | down |
|  | L-PyroglutamicAcid | 1.28E+00 | | 4.46E-01 | down |
|  | Lactose | 1.29E+00 | | 3.58E-01 | down |
|  | Elaidic Acid | 1.28E+00 | | 4.26E-01 | down |
|  | D-Arabitol | 1.26E+00 | | 3.06E+00 | up |
|  | L-Arabitol | 1.33E+00 | | 3.65E+03 | up |
|  | 3-Aminosalicylic acid | 1.30E+00 | | 4.45E-01 | down |
|  | 3-Hydroxybutyrate | 1.27E+00 | | 3.16E-01 | down |
|  | 3,4-Dimethoxyphenyl acetic acid | 1.22E+00 | | 3.88E-01 | down |
|  | 9-(β-D-Arabinofuranosyl)hypoxanthine | 1.30E+00 | | 3.95E+00 | up |
|  | Naringenin chalcone(4,2',4',6'-Tetrahydroxychalcone) | 1.31E+00 | | 1.67E-01 | down |
|  | Aldehydo-D-galacturonate | 1.29E+00 | | 2.53E-01 | down |
|  | Stearic Acid | 1.31E+00 | | 3.62E-01 | down |
|  | Caffeine | 1.29E+00 | | 3.42E-01 | down |
|  | 11-Octadecanoic acid(Vaccenic acid) | 1.29E+00 | | 4.10E-01 | down |
|  | Oxidized Glutathione | 1.31E+00 | | 3.54E-01 | down |
|  | Turanose | 1.29E+00 | | 4.39E+00 | up |
|  | MAG(18:3)isomer5 | 1.19E+00 | | 4.87E-01 | down |
|  | MAG(18:2)isomer1 | 1.27E+00 | | 2.79E-01 | down |
|  | MAG(18:1)isomer2 | 1.24E+00 | | 4.20E-01 | down |
|  | MAG(18:4)isomer3 | 1.31E+00 | | 4.50E-01 | down |
|  | MAG(18:3)isomer3 | 1.26E+00 | | 3.62E-01 | down |
|  | Cocamidopropyl βine | 1.31E+00 | | 2.46E-01 | down |
|  | MAG(18:3)isomer2 | 1.15E+00 | | 4.79E-01 | down |
|  | L-Asparagine Anhydrous | 1.32E+00 | | 2.43E-01 | down |
|  | Syringic acid O-glucoside | 1.30E+00 | | 3.27E+00 | up |
|  | L-Glutamic acid | 1.31E+00 | | 7.01E-02 | down |
|  | L-(+)-Lysine | 1.20E+00 | | 2.58E-01 | down |
|  | L-Glutamine | 1.24E+00 | | 3.21E-01 | down |
|  | 6-Aminocaproic acid | 1.30E+00 | | 4.97E-01 | down |
|  | Ethyl gallate | 1.20E+00 | | 3.27E-01 | down |

| **number** | | **Compounds** | **VIP** | **Fold_Change** | | **Type** |
| --- | --- | --- | --- | --- | --- | --- |
|  | D-(+)-Sucrose | | 1.29E+00 | | 3.70E+00 | up |
|  | Gluconic acid | | 1.32E+00 | | 2.53E-01 | down |
|  | Guanine | | 1.30E+00 | | 2.32E+00 | up |
|  | L-Methionine methyl ester | | 1.25E+00 | | 4.71E-01 | down |
|  | 4-Hydroxy-L-glutamic acid | | 1.22E+00 | | 2.36E+00 | up |
|  | 3,4,5-Trimethoxyphenyl-β-D-Glucopyranoside | | 1.28E+00 | | 2.09E+00 | up |
|  | 1-O-Galloyl-β-D-glucose | | 1.32E+00 | | 1.06E+01 | up |
|  | Galloyl Methyl gallate | | 1.23E+00 | | 2.58E+00 | up |
|  | 1,6-Bis-O-galloyl-β-D-glucose | | 1.29E+00 | | 2.63E+00 | up |
|  | 1,2,3,4,6-Penta-O-galloyl-β-D-glucose | | 1.22E+00 | | 2.54E+00 | up |
|  | Pentagalloylglucose | | 1.19E+00 | | 2.34E+00 | up |
|  | Quercetin-7-O-(6'-O-malonyl)-β-D-glucoside | | 1.06E+00 | | 3.32E+00 | up |
|  | 5-Aminocycloheptane-1,2,3-triol | | 1.29E+00 | | 3.66E+00 | up |
