# Supplementary material for: Analysis of the mechanism of Ricinus communis L. tolerance to Cd metal based on proteomics and metabolomics
Source: PLoS One. 2023 Mar 2;18(3):e0272750. doi: 10.1371/journal.pone.0272750 (PMC9980742; doi:10.1371/journal.pone.0272750)
Supplement: S9 Table — (DOCX) [file pone.0272750.s009.docx]

Table S9 Gene sequence alignment results

Original atggctaagtctatcagtctcgaagagatcaagaatgaaactgtcgatctggaacgtatt 60

Determination atggctaagtctatcagtctcgaagagatcaagaatgaaactgtcgatctggaacgtatt 60

************************************************************

Original cccattgatgaagtgtttgagcaactgaaatgtaccagagaaggtttaagttcagacgaa 120

Determination cccattgatgaagtgtttgagcaactgaaatgtaccagagaaggtttaagttcagacgaa 120

************************************************************

Original ggaaccaaccggcttcaaatctttggacccaacaagctagaagagaaaaaggaaagcaaa 180

Determination ggaaccaaccggcttcaaatctttggacccaacaagctagaagagaaaaaggaaagcaaa 180

************************************************************

Original attctcaagtttttggggttcatgtggaacccactatcatgggtcatggaagctgcagct 240

Determination attctcaagtttttggggttcatgtggaacccactatcatgggtcatggaagctgcagct 240

************************************************************

Original attatggcaattgcgttggcgaatggtgatggagagcccccggattggcaagactttatt 300

Determination attatggcaattgcgttggcgaatggtgatggagagcccccggattggcaagactttatt 300

************************************************************

Original ggtattatttgcttgctggtgatcaactctactatcagtttcattgaagagaacaatgct 360

Determination ggtattatttgcttgctggtgatcaactctactatcagtttcattgaagagaacaatgct 360

************************************************************

Original ggcaatgctgctgctgcccttatggctggtctggctcctaaaacaaaggtgcttagagat 420

Determination ggcaatgctgctgctgcccttatggctggtctggctcctaaaacaaaggtgcttagagat 420

************************************************************

Original ggcaaatggactgagcaggaagctgcaatcctggttccaggagatatcattagtatcaaa 480

Determination ggcaaatggactgagcaggaagctgcaatcctggttccaggagatatcattagtatcaaa 480

************************************************************

Original ttgggagatattgttcctgctgatgctcgtcttcttgagggtgatccgttaaagattgat 540

Determination ttgggagatattgttcctgctgatgctcgtcttcttgagggtgatccgttaaagattgat 540

************************************************************

Original caatctgccctcactggagaatcactgccagtgaccaagaatcctggtgatgaagttttc 600

Determination caatctgccctcactggagaatcactgccagtgaccaagaatcctggtgatgaagttttc 600

************************************************************

Original tctggttccacttgcaaacaaggagaaattgaggctgtcgttatagccactggtgttcac 660

Determination tctggttccacttgcaaacaaggagaaattgaggctgtcgttatagccactggtgttcac 660

************************************************************

Original accttcttcggaaaggctgcacatcttgtggacagcaccaacaatgttgggcacttccag 720

Determination accttcttcggaaaggctgcacatcttgtggacagcaccaacaatgttgggcacttccag 720

************************************************************

Original aaagtgctcactgctatcgggaacttctgtatttgttctatagcaattggaatgttggtt 780

Determination aaagtgctcactgctatcgggaacttctgtatttgttctatagcaattggaatgttggtt 780

************************************************************

Original gaaatcatagtcatgtaccctattcagcaccgcaagtacagggatggaattaacaatctc 840

Determination gaaatcatagtcatgtaccctattcagcaccgcaagtacagggatggaattaacaatctc 840

************************************************************

Original ttggttcttttgattggtggtatcccaattgctatgccaacagtcttgtctgttacaatg 900

Determination ttggttcttttgattggtggtatcccaattgctatgccaacagtcttgtctgttacaatg 900

************************************************************

Original gccattgggtctcacaagttgtctcaacaaggggccatcactaagcgtatgactgccatt 960

Determination gccattgggtctcacaagttgtctcaacaaggggccatcactaagcgtatgactgccatt 960

************************************************************

Original gaagaaatggcaggtatggatgtcctttgcagtgacaaaactggcacccttaccctcaac 1020

Determination gaagaaatggcaggtatggatgtcctttgcagtgacaaaactggcacccttaccctcaac 1020

************************************************************

Original aagctgagtattgacaaagccttgattgaggtctttgcaaagggtgttgaaaaagatcac 1080

Determination aagctgagtattgacaaagccttgattgaggtctttgcaaagggtgttgaaaaagatcac 1080

************************************************************

Original gtgatactgcttgctgcaagggcctctagagttgagaatcaggatgctattgatgctgcc 1140

Determination gtgatactgcttgctgcaagggcctctagagttgagaatcaggatgctattgatgctgcc 1140

************************************************************

Original atggttggaatgctagctgatcctaaagaggcaagagctggtataagagaggtgcacttc 1200

Determination atggttggaatgctagctgatcctaaagaggcaagagctggtataagagaggtgcacttc 1200

************************************************************

Original ttgccattcaaccctgtggacaagaggactgctttgacttatatcgatgctgatggcaac 1260

Determination ttgccattcaaccctgtggacaagaggactgctttgacttatatcgatgctgatggcaac 1260

************************************************************

Original tggcaccgtgccagtaaaggtgctcctgagcagatcttgagcttgtgcaattccagggaa 1320

Determination tggcaccgtgccagtaaaggtgctcctgagcagatcttgagcttgtgcaattccagggaa 1320

************************************************************

Original gatctgaagaggaaagttcattcagttatagataaatttgctgaacgtgggctacgatca 1380

Determination gatctgaagaggaaagttcattcagttatagataaatttgctgaacgtgggctacgatca 1380

************************************************************

Original ttggctgtggctagacagcaagttcctgagaaaaacaaagacagtccaggtagtccatgg 1440

Determination ttggctgtggctagacagcaagttcctgagaaaaacaaagacagtccaggtagtccatgg 1440

************************************************************

Original gagtttgtcggcttgttgaacctctttgatcctcccaggcatgacagtgcagaaacaatc 1500

Determination gagtttgtcggcttgttgaacctctttgatcctcccaggcatgacagtgcagaaacaatc 1500

************************************************************

Original cgcagggctcttaaccttggtgtcaatgtcaagatgattactggtgatcaacttgctatt 1560

Determination cgcagggctcttaaccttggtgtcaatgtcaagatgattactggtgatcaacttgctatt 1560

************************************************************

Original gcaaaggagactgggagaagacttggtatgggaaccaacatgtacccatctgctacattg 1620

Determination gcaaaggagactgggagaagacttggtatgggaaccaacatgtacccatctgctacattg 1620

************************************************************

Original cttggtcaggacaaggatgcaagcattgctgctcttcctgtagaagagttgattgagaag 1680

Determination cttggtcaggacaaggatgcaagcattgctgctcttcctgtagaagagttgattgagaag 1680

************************************************************

Original gctgatggttttgccggcgtgtttccagagcacaaatatgaaatcgtgaagaagctgcaa 1740

Determination gctgatggttttgccggcgtgtttccagagcacaaatatgaaatcgtgaagaagctgcaa 1740

************************************************************

Original gaaagaaagcacattgttggaatgactggcgatggtgttaatgatgctcctgctttgaag 1800

Determination gaaagaaagcacattgttggaatgactggcgatggtgttaatgatgctcctgctttgaag 1800

************************************************************

Original aaggcagatatcggtattgctgttgctgatgctacagatgctgcacgaggtgcttctgac 1860

Determination aaggcagatatcggtattgctgttgctgatgctacagatgctgcacgaggtgcttctgac 1860

************************************************************

Original attgttctaacagaacctggattgagtgttattatcagtgccgtcctgactagcagagct 1920

Determination attgttctaacagaacctggattgagtgttattatcagtgccgtcctgactagcagagct 1920

************************************************************

Original attttccaaagaatgaagaattatactatttatgctgtttccatcacaatccgtattgtg 1980

Determination attttccaaagaatgaagaattatactatttatgctgtttccatcacaatccgtattgtg 1980

************************************************************

Original tttggtttcatgcttattgctctgatatggcagtatgacttctctcctttcatggttttg 2040

Determination tttggtttcatgcttattgctctgatatggcagtatgacttctctcctttcatggttttg 2040

************************************************************

Original attattgctatcctaaatgacggaacaattatgacaatctcaaaggatagagtaaagcca 2100

Determination attattgctatcctaaatgacggaacaattatgacaatctcaaaggatagagtaaagcca 2100

************************************************************

Original tcacccttgcctgatagctggaaattaaaagagatttttgccactggaattgtgcttggc 2160

Determination tcacccttgcctgatagctggaaattaaaagagatttttgccactggaattgtgcttggc 2160

************************************************************

Original ggctacttggcactgatgactgttatattcttctgggccatgcatgataccgacttcttt 2220

Determination ggctacttggcactgatgactgttatattcttctgggccatgcatgataccgacttcttt 2220

************************************************************

Original tctgacaaatttggtgtaagatctatacgtagaagtgaccctgaattgatgggtgcttta 2280

Determination tctgacaaatttggtgtaagatctatacgtagaagtgaccctgaattgatgggtgcttta 2280

************************************************************

Original taccttcaagtcagtatcgtgagccaggctctcatttttgttactcggtctcgcagctgg 2340

Determination taccttcaagtcagtatcgtgagccaggctctcatttttgttactcggtctcgcagctgg 2340

************************************************************

Original tcttattttgaacgtcctggcctgctactggtgactgctttcatcattgcacaactggtt 2400

Determination tcttattttgaacgtcctggcctgctactggtgactgctttcatcattgcacaactggtt 2400

************************************************************

Original gcaactttgattgccgtatatgctaactggggctttgcacacatcaagggaattggctgg 2460

Determination gcaactttgattgccgtatatgctaactggggctttgcacacatcaagggaattggctgg 2460

************************************************************

Original ggatgggctggtgttatctggctttacagtattgttttctatatcccactcgacttgctg 2520

Determination ggatgggctggtgttatctggctttacagtattgttttctatatcccactcgacttgctg 2520

************************************************************

Original aagttcgccattcgttacatcctcagtggaaaggcttggctcaacttgctagagaacaaa 2580

Determination aagttcgccattcgttacatcctcagtggaaaggcttggctcaacttgctagagaacaaa 2580

************************************************************

Original actgcattcaccaccaagaaagattatggtaaagaggaaagagaagctcaatgggctctt 2640

Determination actgcattcaccaccaagaaagattatggtaaagaggaaagagaagctcaatgggctctt 2640

************************************************************

Original gctcaaaggaccttgcatggacttcaaccaccagaaactgccagtatcttcaacgaaaag 2700

Determination gctcaaaggaccttgcatggacttcaaccaccagaaactgccagtatcttcaacgaaaag 2700

************************************************************

Original agcagctacagagagttgtctgagatcgctgagcaggccaagagacgagccgaggttgca 2760

Determination agcagctacagagagttgtctgagatcgctgagcaggccaagagacgagccgaggttgca 2760

************************************************************

Original aggcttcgggagcttcacaccctaaagggacatgttgaatcagtggttaagcttaagggc 2820

Determination aggcttcgggagcttcacaccctaaagggacatgttgaatcagtggttaagcttaagggc 2820

************************************************************

Original ttggacattgatacaattcagcagcattatacagtgtaa 2859

Determination ttggacattgatacaattcagcagcattatacagtgtaa 2859

***************************************
