# Supplementary material for: Analysis of the mechanism of Ricinus communis L. tolerance to Cd metal based on proteomics and metabolomics
Source: PLoS One. 2023 Mar 2;18(3):e0272750. doi: 10.1371/journal.pone.0272750 (PMC9980742; doi:10.1371/journal.pone.0272750)
Supplement: Attachment — Submitted filename: Response to Reviewers-10.18.docx [file pone.0272750.s011.docx]

**Response to Reviewer #1**

1.The title of the paper has been modified.

2.Data from the study have been added to the abstract.However, THERE ARE not MANY changes. I think enough content has been added to this abstract. The omics content is more complicated and too much has been added to the abstract. I need you to see if it meets the requirements.

3.The key word question has been revised as requested.

7.Castor oil is obtained from the seeds of the castor plant. Castor oil is a non-edible oil, which can lead to poisoning if consumed in large quantities. However, in traditional Chinese medicine, a small amount of castor oil can be used to treat gastrointestinal diseases.

9.The physichochemical properties of sowing media have been added to the article.

10.Because the method used in the experiment is soil culture, not medium culture method, so, the seeds in this part do not need to be sterilized.

11.The container material, size and sand content used in the experiment have been added to the paper.

12-16.The text has been amended in accordance with the proposed amendments, But there may be a deviation from the position of the Reviewer mark, If the modification is not sufficient, I'm willing to revise it, thanks.

17.I have revised some contents of the discussion and references, but most of them cannot be replaced, so I need your understanding.

18.I have reduced and revised the conclusions as requested.

**Response to Reviewer #2**

Sorry, I can only see your annotations in the PDF, but I don't see relevant notes, so I can only modify by myself. If the modification does not meet your expectations, I need you to contact me to modify it again.
